# Upregulated NOTCH2 Expression Is Implicated in the Clinical Aggressiveness of Atypical Fibroxanthoma and Pleomorphic Dermal Sarcoma

**DOI:** 10.1111/ijd.17844

**Published:** 2025-05-19

**Authors:** Yi‐Pei Lee, Fahimeh Razegphpour, Emilia Sorescu, Markus Stücker, Thilo Gambichler

**Affiliations:** ^1^ Department of Dermatology, Venerology and Allergology St. Josef Hospital, Ruhr‐University Bochum Bochum Germany; ^2^ Department of Dermatology, Dortmund Hospital gGmbH University Witten/Herdecke Germany

**Keywords:** atypical fibroxanthoma, HES1, NICD, NOTCH1, NOTCH2, Notch‐targeted therapy, pleomorphic dermal sarcoma

## Abstract

**Background:**

Atypical fibroxanthoma (AFX) and pleomorphic dermal sarcoma (PDS) represent clinicopathological variants of a spectrum. It is known that AFX and PDS tumors harbor frequent NOTCH1/2 mutations. However, the expression of Notch signaling pathway‐associated proteins in both tumor cells has not been studied before.

**Methods:**

We conducted an immunohistochemical study by performing NOTCH1, NOTCH2, NICD, and HES1 staining on the most representative formalin‐fixed paraffin‐embedded (FFPE) tumor tissues out of sixty‐two patients with the first diagnosis of either AFX (*n* = 33) or PDS (*n* = 29) in a single tertiary medical center.

**Results:**

Ten patients (PDS, *n* = 9; AFX, *n* = 1) had disease progression in terms of locoregional relapse, including local recurrence and regional lymph node metastasis, with a median time‐to‐(first)‐recurrence interval of 8 months after a wide local excision. Among all the Notch expression profiles, only the upregulated NOTCH2 expression has a positive correlation with disease progression [odds ratio (OR): 1.02, 95% confidence interval (CI): 1–1.04, *p* = 0.029]. Furthermore, HES1 is activated through the NOTCH2 signaling pathway (*r* (60) = 0.27, *p* = 0.032) rather than NOTCH1, and NOTCH1 does not appear to be functionally active.

**Conclusions:**

Upregulated NOTCH2 expression plays a significant role in disease progression, in part through the canonical signaling pathway involving the downstream effector HES1. Targeting NOTCH2 signaling might hold therapeutic promise in patients with disease progression.

## 1 Introduction

Atypical fibroxanthoma (AFX) and pleomorphic dermal sarcoma (PDS) are cutaneous fibrohistiocytic tumors representing part of a spectrum according to similar clinical, histological, genetic, and epigenetic features [1]. PDS has a more aggressive biological behavior, which is defined by the presence of at least one of the following histopathological features: infiltration into subcutaneous fat, perineural/lymphovascular invasion, and presence of tumor necrosis [2]. Both AFX and PDS have a high number of similar recurrent mutations in multiple genes, including FAT1, NOTCH1/2, CDKN2A, TP53, and the TERT promoter [3]. This strongly supports the contention that these two neoplasms are part of the same spectrum. Among others, AFX and PDS harbor frequent NOTCH1/2 mutations, most of which are missense and nonsense mutations [4]. As far as we know, there are no other molecular or genetic studies with regard to prognostic biomarkers for AFX and PDS. However, a recent study has identified prognostic biomarkers in undifferentiated pleomorphic sarcoma (UPS) [5]. UPS is considered to be related to AFX and PDS, with the worst prognosis.

The Notch signaling cascade is known to be implicated in numerous aspects of cancer biology, including the maintenance of cancer stem‐like cells, tumor angiogenesis, metastasis, and tumor immune evasion [6, 7, 8]. Large sequencing efforts of cancer genomes have uncovered both gain‐ and loss‐of‐function mutations in different genes involved in the Notch signaling cascade. This indicates that Notch can be both oncogenic and tumor suppressive. Such a binary function appears to be context‐ and tissue‐specific [9, 10, 11]. In clinical application, antibodies targeting Notch receptors (e.g., tarextumab) and ligands (e.g., tarlatamab or rovalpituzumab) are currently being investigated for several tumor entities. However, to the best of our knowledge, Notch expression profiles in AFX and PDS and their relationship with disease progression have never been studied before. Thus, we aim to explore potential therapeutic options for aggressive AFX and PDS, which cannot be adequately managed through surgical excision alone.

**FIGURE 1:**
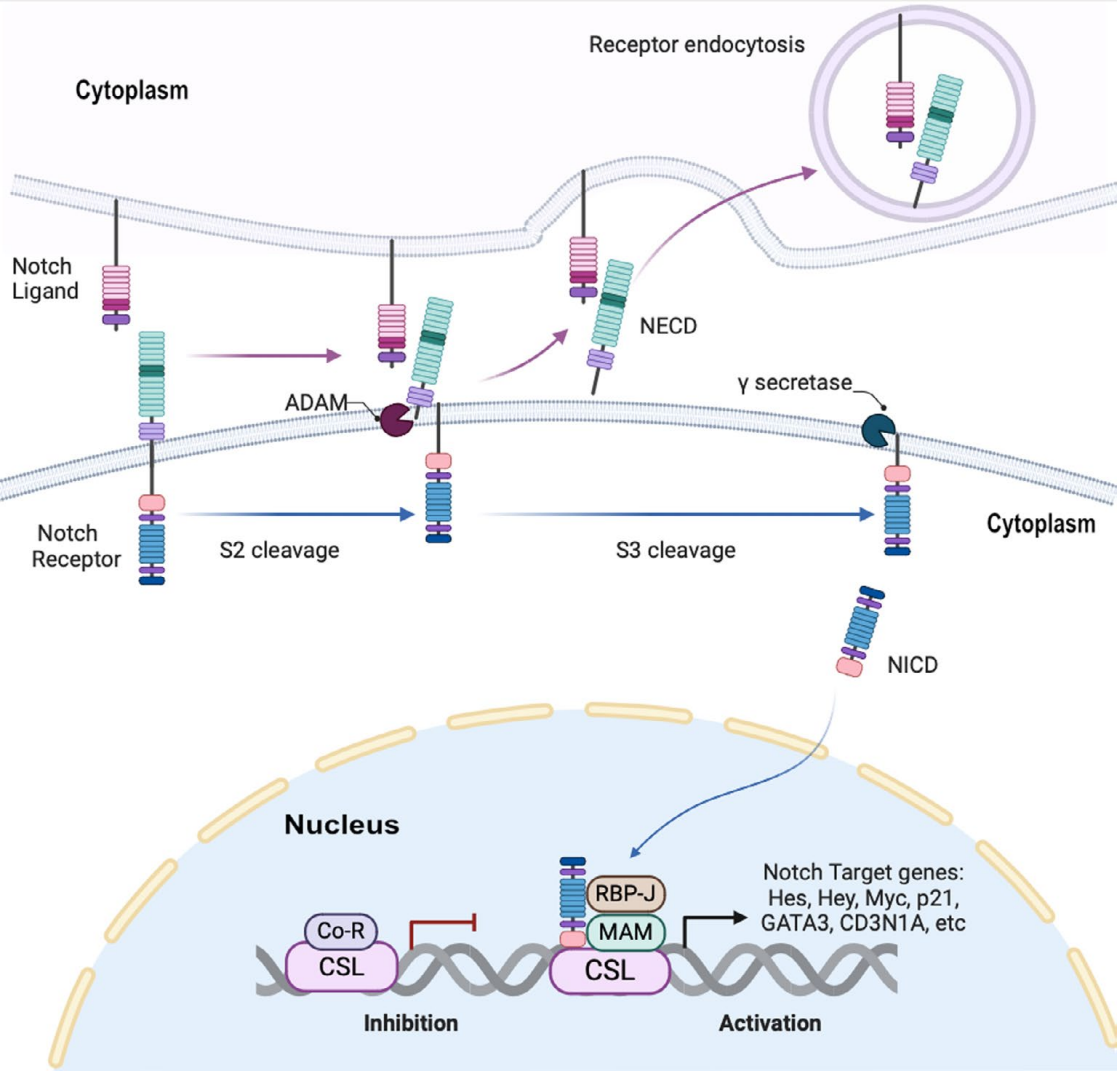
Notch receptor is a type I transmembrane protein. Notch signaling pathway is either initiated by the ligand‐binding via the adjacent signal‐sending cells with the extracellular segments of Notch receptors (NECD) or activated by ligand‐independent Notch receptors. Either way causes NECD to dissociate from its transmembrane subunits and subsequently release the activated intracellular domain of Notch receptors (NICD) via γ‐secretase complex. The activated NICD enters the nucleus and interacts with CSL (CBF1, Suppressor of Hairless, Lag‐1) transcription factor, complexing with other proteins [e.g., recombination signal binding protein for immunoglobulin kappa (κ) J region (RBP‐J) and mastermind‐like (MAML)] to form a transcriptional activation complex, which regulates downstream gene transcription (e.g., of HES1 and Hey1 genes). In the absence of NICD, CSL functions otherwise as a sequence‐specific repressor. The illustration was created with BioRender.com.

The three major components of the Notch signaling pathway in mammals comprise (1) Notch receptors (NOTCH1, NOTCH2, NOTCH3, and NOTCH4); (2) ligands for binding: Jagged1, Jagged2, Delta‐like1/DLL1, DLL3, and DLL4; (3) RBP‐J‐dependent canonical or RBP‐J‐independent non‐canonical downstream effectors [12]. In a canonical pathway, Notch signaling is initiated by ligand binding via the adjacent signal‐sending cells. Subsequently, the activated intracellular domain of Notch receptors (NICD) is released. As a next step, NICD enters the nucleus and interacts with the CSL (CBF1, Suppressor of Hairless, Lag‐1) transcription factor, complexing with other proteins to form a transcriptional activation complex, which regulates downstream gene transcription, e.g., of the HES1 gene. In the absence of NICD, CSL functions otherwise as a sequence‐specific repressor. This canonical pathway is well illustrated in Figure 1.

Herein, we selected two representative Notch receptors (NOTCH1 and NOTCH2), NICD (NOTCH1 intracellular domain, also known as activated NOTCH1), and HES1 (downstream effector) as targets and conducted an immunohistochemical study on tumor samples. Notch expression profiles and clinical outcomes were correlated. The aim of the study was to determine if Notch serves as a prognostic biomarker and to gain a better understanding of its pathway and binary functions involved in AFX and PDS.

## 2 Materials and Methods

### 2.1 Patients and Study Samples

**FIGURE 2:**
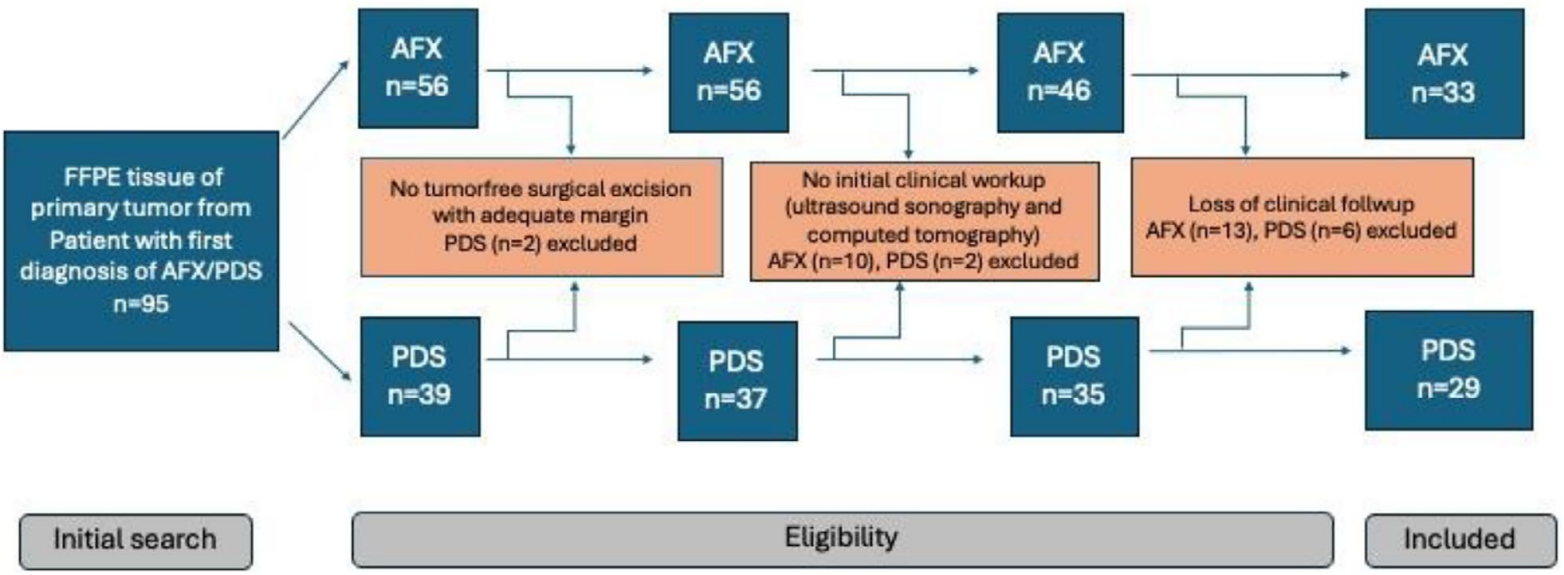
Initial screening and exclusion criteria to identify the eligible participants in the research study.

This retrospective study was conducted in accordance with the STROBE/RECORDs guidelines in a single tertiary medical center in Germany. We chose the most representative formalin‐fixed paraffin‐embedded (FFPE) primary tumor tissues from patients with a first diagnosis of either AFX or PDS from August 1999 to May 2021. Only patients who had sufficient clinical follow‐up data were recruited into this study. Sufficient clinical follow‐up data were obtained until February 2024. In order to exclude lymph node or internal organ involvement, a complete clinical work‐up including ultrasound sonography and computed tomography was performed in every case, and the results were well documented at the time of first diagnosis. The clinical follow‐up data were obtained from medical charts, patient contact, and primary care physicians. The exclusion criteria and eligible study sample for final analysis are illustrated in Figure 2.

### 2.2 Microscopic Workup

AFX and PDS were diagnosed by at least two experienced dermatopathologists, one of whom is a co‐author (M.S.). The diagnosis was made according to an initial morphological examination by routine hematoxylin–eosin staining and exclusion of other neoplasms by vast immunohistochemical staining. The routinely used immunohistochemical panel for AFX/PDS was CD10, vimentin, CD68, MNF116, CK5/6, melan‐A, S‐100, SMA, desmin, CD31, and CD34. The subclassification into AFX and PDS categories was based on the presence of at least one of the following features: infiltration into the subcutaneous fat, perineural/lymphovascular invasion, and presence of tumor necrosis.

The pathological diagnostic approach for AFX/PDS was modified in 2002, following the updated World Health Organization (WHO) criteria [13]. In our study, only one patient was first diagnosed with PDS before 2002. The patient experienced four local recurrences, with the most recent occurring in 2002 when the diagnosis was reconfirmed using the modified WHO criteria.

### 2.3 Immunohistochemistry Staining With NOTCH1, NOTCH2, HES1, and NICD on FFPE Tumor Samples

The immunohistochemistry (IHC) staining of NOTCH1, NOTCH2, NICD, and HES1 was performed following the manufacturer's (Abcam, Cambridge, UK) recommendations and previous papers on FFPE tumor specimens. The FFPE blocks were cut into 4 μm sample sections and mounted on DAKO Microscope Slides (Agilent, Hamburg, Germany). After drying overnight at 37°C, sections of FFPE tissues were deparaffinized in rotihistol (10 min, RT, two times) and subsequently hydrated through a graded alcohol series. For antigen retrieval, sections were heated in Target Retrieval Solution (S2369; Dako Agilent, Hamburg, Germany) for 20 min in a steamer. After sample cooling and a brief washing in Wash Buffer (S330630; Dako Agilent), sections were incubated for 15 min in 1.5% casein in PBS and then blocked for 30 min with Dual Endogenous Enzyme Block at RT. The commercially available antibodies used in this study included rabbit polyclonal anti‐NOTCH1 antibody (ab27526), rabbit polyclonal anti‐NOTCH2 antibody (ab8926), rabbit monoclonal anti‐HES1 antibody (ab108937), and rabbit polyclonal anti‐activated‐NOTCH1 antibody (ab52301). This anti‐activated‐NOTCH1 antibody detects endogenous levels of fragments of activated NOTCH1 resulting from cleavage adjacent to Val1744, thus highlighting NICD. The sample sections were incubated with primary antibodies at different dilutions: NOTCH1 (1:100), NOTCH2 (1:250), HES1 (1:200), and activated‐NOTCH1 (1:100), but under the same pH (9). For a negative control, sample sections were incubated without using a primary antibody. Afterwards, the sample sections were incubated with secondary antibodies by using goat anti‐rabbit antibody or goat anti‐mouse antibody. The biotinylated secondary antibody was detected via incubation with streptavidin‐alkaline phosphatase and treatment with chromogenic substrate (K5005; Dako Agilent; Santa Clara, CA, USA). For nuclear counterstaining, sections were incubated in hematoxylin (S202084; Dako Agilent) for 1 min, followed by a 5‐min incubation in tap water. Finally, the sample sections went through a series of incubations with ascending alcohol concentrations and were mounted with rapid medium for microscopy, Entellan (Merck, Darmstadt, Germany).

### 2.4 Microscopic Evaluation on IHC Staining Result

For microscopic evaluation, slides were scanned at 200x magnification using the Nanozoomer (Hamamatsu, Herrsching am Ammersee, Germany), and the images were evaluated using the viewer software NDP.view2 (Hamamatsu Photonics). Protein expression was evaluated by using histology (H)‐score quantification at a 400x magnification, which was performed by multiplying the staining intensity (1–3) by the percent of positive tumor cells (0–100) in relation to all tumor cells in a single field, yielding a range of 0 to 300. In each section, five fields were chosen randomly and equally distributed from the center to the periphery of the tumor mass. The final H‐score result came out of the sum of the H‐score in each field divided by 5. The tumor cells were considered positive for staining in a membranous/cytoplasmic/nuclear pattern with NOTCH1 and NOTCH2 and in a nuclear pattern with NICD and HES1.

### 2.5 Outcome

Any AFX/PDS‐related event, such as locoregional relapse (local recurrence and regional lymph node metastasis), distant metastasis, and death, will be considered as an event of interest (EOI). If there are multiple EOI in a single patient, only the time‐to‐first EOI is relevant for statistical analysis. All the non‐AFX/PDS‐related deaths, e.g., acute myocardial infarction, will be considered a competing event. Censoring for loss of follow‐up is defined either by the last time in a patient encounter (measured event) or an observable outcome outside a patient encounter (captured event), e.g., follow‐up information through contact with the primary care physicians.

### 2.6 Statistical Analysis

In our study, data were analyzed by using an independent sample two‐tailed t‐test, chi‐squared test, Spearman correlation, and logistic regression with odds ratio (OR) and 95% confidence interval (CI) given. In order to illustrate the EOI survival rate and censored data, a stratified cause‐specific Cox regression was used and plotted under the Kaplan–Meier survival curve by the “ggsurvplot” command in R software. Furthermore, a cumulative incidence function was used to estimate the risk of different events over a period of time by the “ggcompetingrisks” command. A *p*‐value < 0.05 was considered significant.

## 3 Results

### 3.1 Patient Characteristics and Clinical Data

Sixty‐two patients were eligible for final analysis, which included AFX (*n* = 33) and PDS (*n* = 29). The median follow‐up period was 45.5 months. Of the 62 patients, 50 were males and 12 were females. The patients were aged between 40 and 94 years (median age 79) at the time of first diagnosis. Nearly all of the primary tumors (95.2%) occurred exclusively in the head and neck area. Of the three cases with primary tumors outside the head and neck area, one occurred on the trunk (PDS), one on the upper extremity (AFX), and another on the lower extremity (AFX). All of the tumors were excised with wide local excision. The surgical margin was 0.5–1 cm for AFX and 1–2 cm for PDS. None of the patients had lymph node or internal organ metastasis at the time of diagnosis, and none of the patients ever received adjuvant radiotherapy or chemotherapy. In the follow‐up period, there was at least one local recurrence in 9 patients (PDS, *n* = 8; AFX, *n* = 1). One PDS patient developed a regional lymph node metastasis (metastasis to the parotid gland) in the follow‐up period of 12 months. Two PDS patients had multiple recurrences (at least three times). The median time‐to‐(first)‐recurrence interval was 8 months (range: 4–138 months). None of the above patients who developed either recurrence or metastasis had a known concurrent or pre‐existing immunocompromised status. None of the AFX patients developed metastasis. However, two AFX patients had a second primary tumor outside the locoregional area within 14 and 21 months, respectively. Over the follow‐up period, 10 patients died of non‐AFX/PDS‐related causes. Among them, one PDS patient died of impaired wound healing within 6 months of the last excisional surgery after the fourth recurrence. Otherwise, none of the patients died of AFX or PDS. The basic data is illustrated in Table 1.

**TABLE 1:** Basic data, clinical outcomes, and Notch‐associated protein expression in patients with a first diagnosis of atypical fibroxanthoma (AFX) or pleomorphic dermal sarcoma (PDS) (*n =* 62).

	PDS (*n* = 29)	AFX (*n* = 33)
Basic data		
Age at diagnosis in years^a^ (range)	79 (58–94)	80 (40–93)
Gender: male/female—no. (%)	26/3 (89.7/10.3)	24/9 (72.7/27.3)
Location on head and neck area—no. (%)	28 (96.6)	31 (93.9)
Follow‐up period in months^a^ (range)	54 (3–181)	42 (1–137)
Clinical outcomes		
Event of interest: recurrence/metastasis—patient no. (%)	8 (27.6)/1 (3.4)	1 (3.0)/0 (0)
Time‐to‐first‐event interval in months^a^ (range)	9 (4–138)	6
Second primary tumor—patient no. (%)	0 (0)	2 (6.1)
Time to occurrence of second primary tumor in months^a^ (range)	N/A	17.5 (14–21)
Death—no. (%)	0 (0)	0 (0)
Overall follow‐up period in months^a^ (range)	54 (3–181)	42 (1–137)
Expression of Notch‐associated protein in H‐score^c^		
NOTCH1^b^ (SD)	84.7 (33.1)	89.2 (32.4)
NOTCH2^b^ (SD)	125.2 (50.7)	95.2 (38.9)
HES1^b^ (SD)	119.3 (47.1)	111.1 (37.7)
NICD^b^ (SD)	0 (0)	0 (0)

Abbreviations: N/A, not applicable; SD, standard deviation.

^a^ Non‐normally distributed data expressed with median and range.

^b^ Normally distributed data expressed with mean and standard deviation (SD).

^c^ H‐score ranging from 1 to 300.

Our study considered disease progression in terms of local recurrence and regional lymph node metastasis to be the same EOI. Even though there could be more than one EOI in the same patient, the subsequent EOI other than the first EOI did not generate additional value, because the IHC study was only performed on the first primary tumor. A second primary tumor was seen in two AFX patients, which was not considered an EOI because it is not an index for the biological behavior of the first primary tumor.

**FIGURE 3:**
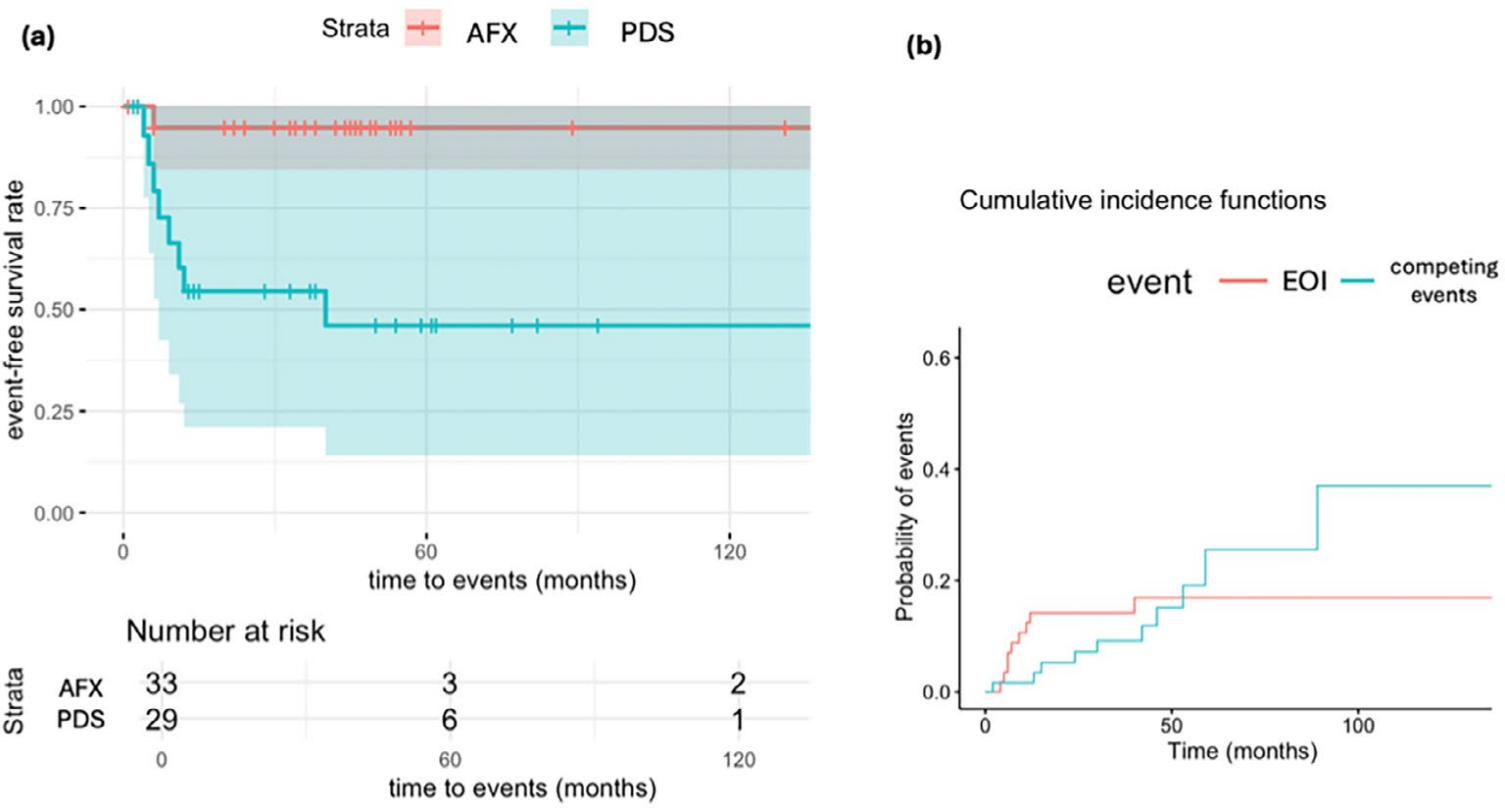
(a) Stratified cause‐specific Cox‐regression model plotted by Kaplan–Meier survival curves and risk table show the EOI‐free survival in relation with the time‐to‐(first)‐event interval after the first diagnosis of either AFX or PDS. Notably, we had 10 competing events, which are considered censored by the Cox proportional hazards model. (b) Cumulative incidence function estimates the risk in different events over a period of time: EOI (AFX/PDS‐related events) and competing events (non‐AFX/PDS‐related deaths). Due to a certain amount of censored data (including competing events) within the follow‐up period, the EOI‐free survival rate might be biased, either overestimated or underestimated. EOI, event of interest.

To better visualize the clinical outcomes and EOI between the AFX and PDS groups in relation to the follow‐up period, the data were plotted by Kaplan–Meier survival curves (Figure 3a). The 5‐year estimate for EOI is 17.48% (Figure 3b). The binary logistic regression analysis showed that PDS tumors are associated with a significant disease progression/unfavorable clinical outcome (*p* = 0.015, OR = 14.4, 95% CI = 1.69–122.42).

### 3.2 Results of Immunohistochemistry Study of NOTCH1, NOTCH2, HES1, and NICD

**FIGURE 4:**
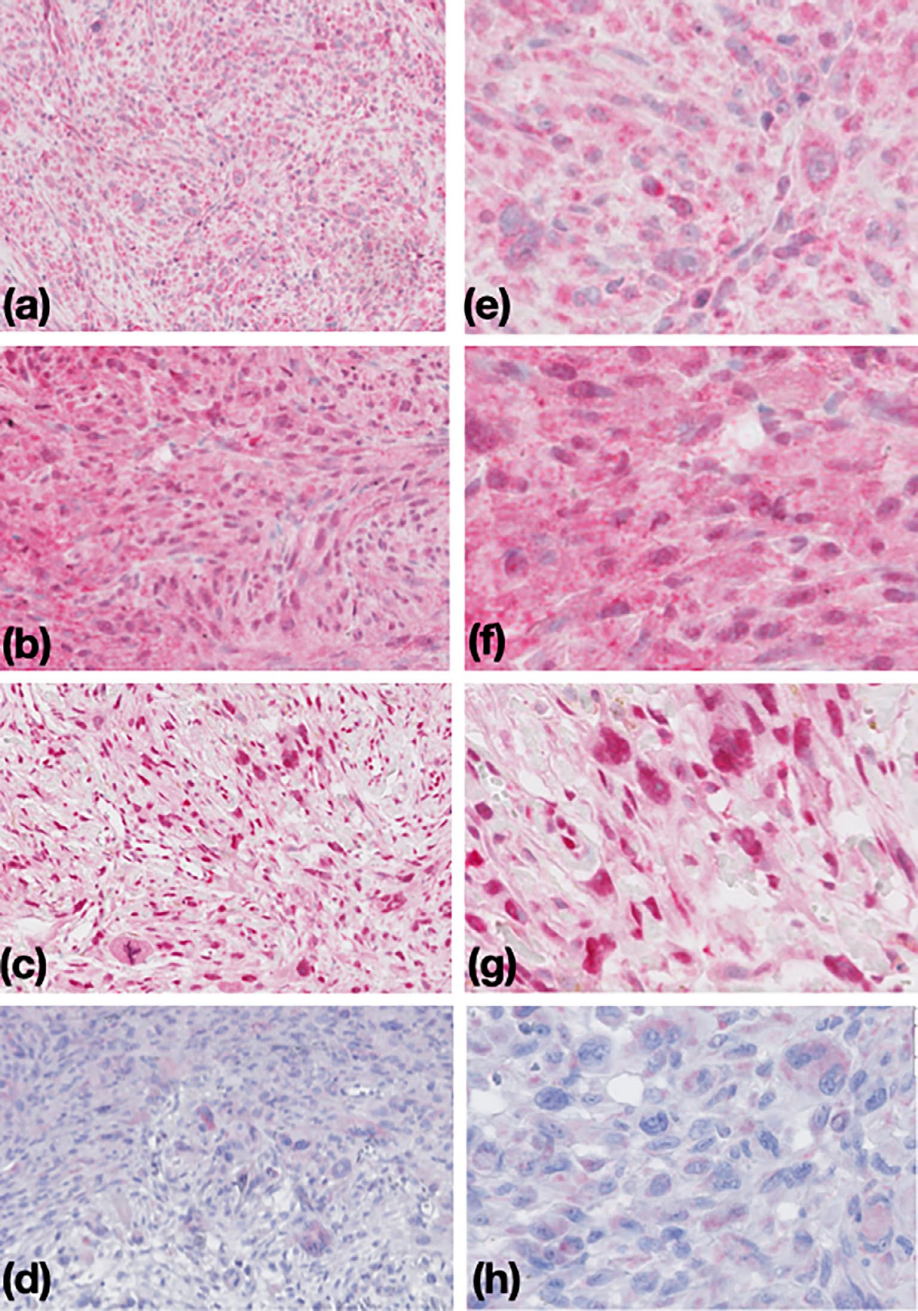
(a–d) at 200x magnification, (e–h) at 400x magnification. NOTCH1 (a and e) immunohistochemistry (IHC) staining showed a predominantly cytoplasmic pattern, and NOTCH2 (b and f) showed both cytoplasmic and nuclear patterns. HES1 (c and g) was not restrictively expressed in the nucleus but also in the cytoplasm. NICD (d and h) was not expressed in the nucleus at all, and although there was a weak cytoplasmic staining, it was regarded as negative.

Both NOTCH1 and NOTCH2 were expressed in AFX and PDS tumor cells, and the staining showed mostly a cytoplasmic pattern with or without a nuclear pattern. Nuclear staining of NICD was not observed in either PDS or AFX tumor cells, thus being considered negative. HES1, a downstream effector and surrogate marker for active Notch, was not only present in the nucleus but also in the cytoplasm in some of the tumor cells (Figure 4).

To determine whether there was a significant difference in each Notch gene expression profile (NOTCH1, NOTCH2, and HES1) between the PDS and AFX groups, we used the two‐tailed t‐test for independent samples. Before starting data analysis, it was proved that the protein expression of NOTCH1, NOTCH2, and HES1 assessed by H‐score (range 0–300) followed a normal distribution.

In addition, we observed a discordant degree of expression between NOTCH1 and HES1 in the same tumor samples. Due to this reason, we wanted to know if HES1 is concordant with NOTCH2 expression. HES1 is not only a well‐known downstream effector of NOTCH1 but also of NOTCH2 via the same CSL binding site in a canonical pathway. Therefore, we performed a Pearson correlation analysis to determine whether HES1 activation occurred through the NOTCH1 or NOTCH2 signaling pathway. It showed that there was a statistically significant positive correlation between NOTCH2 and HES1 (*p* = 0.032), but not between NOTCH1 and HES1. It may indicate that HES1 was, at least, partially coupled from NOTCH2 rather than NOTCH1 in AFX and PDS.

### 3.3 Correlation With Clinical Outcome

**FIGURE 5:**
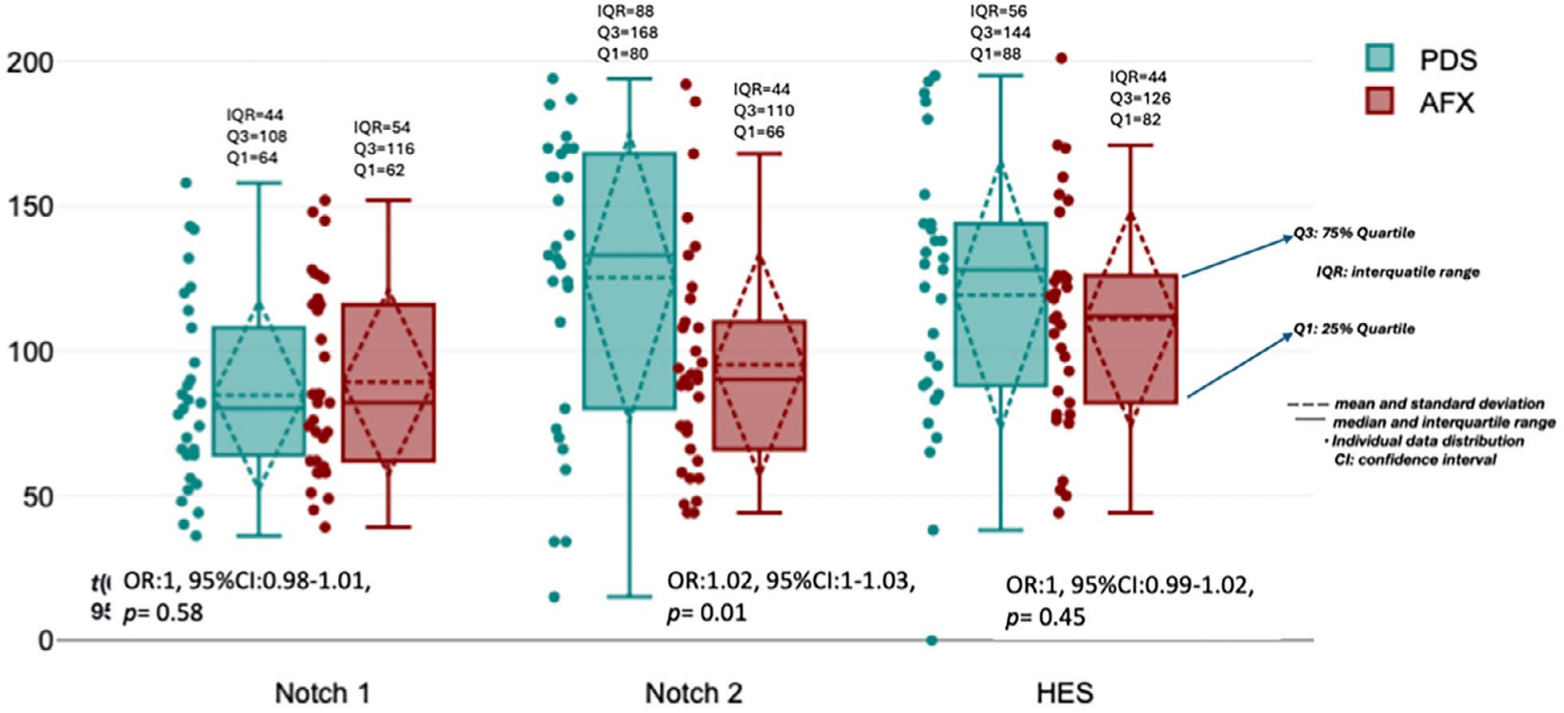
Higher NOTCH2 expression in PDS than in AFX tumors with a statistical significance (*p* = 0.011), and the Cohen's d value 0.67 represented a medium effect size, which indicated an unneglected power of the statistical significance. The data were plotted with Datatab (online statistic software, Graz, Austria).

Among all the Notch expression profiles, only NOTCH2 had a statistically significantly higher expression/upregulation in PDS than AFX tumor cells (OR: 1.02, 95% CI: 1–1.03, *p* = 0.01) (Figure 5). The upregulated NOTCH2 expression had a statistically significant positive correlation with disease progression (OR: 1.02, 95% CI: 1–1.04, *p* = 0.029), whereas neither NOTCH1 nor HES1 expression showed a relationship with disease progression (OR: 0.99, 95% CI: 0.97–1.01, *p* = 0.365 and OR: 1, 95% CI: 0.98–1.01, *p* = 0.69, respectively).

## 4 Discussion

In our study, although a surgical excision with a wider tumor‐free margin was performed in PDS patients than in AFX patients, disease progression in terms of local recurrence and locoregional lymph node metastasis occurred more frequently in PDS (*n* = 9) than in AFX (*n* = 1) patients. Several systematic reviews and meta‐analyses have demonstrated that Mohs micrographic surgery (MMS) is superior to conventional surgery with wide local exicison (WLE) for the treatment of AFX [14]. Some preliminary studies suggest MMS as a treatment of choice for PDS as well. As an alternative to MMS, a 2‐cm WLE could show a prognostic benefit for PDS [15]. However, strong evidence‐based recommendations for PDS are lacking due to the rarity of this tumor and the paucity of data. Our study supports the previous reports in the literature that PDS has a more aggressive clinical behavior than AFX, and the clinical aggressiveness of PDS could not be combated with a surgical margin of 1–2 cm. Notably, we observed two AFX patients who developed a second primary AFX within 2 years and one PDS patient who had a recurrence after a disease‐free interval of 181 months (15.1 years). Although the development of a second primary tumor or a recurrence more than 10 years after the surgical excision is uncommon, similar cases have been reported [16, 17].

Notch signaling plays a binary role in different cancer types and cells. Activation of the Notch signaling pathway is known to have an oncogenic function in T‐cell acute lymphoblastic leukemia, soft tissue sarcoma, and osteosarcoma [18, 19]. On the other hand, it plays a suppressive role in acute myeloid leukemia, glioma, angiosarcoma, Ewing's sarcoma, and cutaneous squamous cell carcinoma [20, 21, 22]. Therefore, Notch acts in a tissue‐ and context‐dependent manner, thus accounting for functional diversity in carcinogenesis in different tumor types. As we observed in our study that the upregulated NOTCH2 correlated with an unfavorable outcome, we assume that NOTCH2 has an oncogenic function in AFX and PDS.

NOTCH1 and NOTCH2 share the highest homology among other Notch receptors [18]. However, our study showed only an upregulation of NOTCH2 expression in PDS. Even though the tumor cells also expressed NOTCH1, there was no upregulation in either the AFX or the PDS group. In some cancer types, NOTCH2 has shown a significant relevance to aggressiveness and carcinogenesis instead of NOTCH1, such as gastric cancer, hepatic carcinoma, pancreatic cancer, and bladder cancer [23, 24, 25, 26, 27]. Furthermore, in our study, NOTCH1 did not appear to be transcriptionally active in AFX and PDS because NICD was not expressed in the nucleus, and HES1 didn't significantly correlate with NOTCH1. Another example of functionally inactive NOTCH1 with uncoupling HES1 expression was evidenced by cytoplasmic staining in Ewing's sarcoma [28].

Even though HES1 positively correlated with NOTCH2 in our study, HES1 did not show a positive relationship with disease progression. We speculate that there could be other downstream effectors, e.g., Hey1 [27, 28], or a non‐canonical pathway involved by cross‐talking with other signaling pathways, e.g., Hedgehog and Wnt pathways [29, 30]. In addition, it is known that HES1 can be activated by Ras/MAPK signaling without Notch activation [31]. Therefore, HES1 may not be the best surrogate marker for the Notch signaling pathway, at least for AFX and PDS tumor cells. Further study by using anti‐activated NOTCH2 antibodies for intracytoplasmic segments of NOTCH2 receptors might provide us with more information.

## 5 Limitation

The limitation of this study lies in the difficulty in collecting standardized follow‐up data; notably, a certain number of patients were censored within a 5‐year follow‐up period. Furthermore, we didn't perform genetic testing for correlation in order to make sure that all the AFX and PDS included in this study didn't have other significant mutational burdens and copy number changes. We could not exclude pathways other than Notch, which play a more critical role in the disease progression among AFX and PDS.

## 6 Conclusions

In our study, we were able to prove that an upregulated NOTCH2 expression is significantly implicated in disease progression in terms of locoregional relapse (local recurrence and regional lymph node metastasis), especially in PDS. The downstream effector HES1 was activated through NOTCH2 rather than NOTCH1 upregulation. Whether crosstalk with other signaling pathways or downstream effectors other than HES1 were involved in NOTCH2 signaling is still elusive. Further studies on NOTCH2 signaling pathway‐associated proteins might shed light on this. NOTCH2‐targeted immunotherapy may be a rational novel treatment strategy for aggressive AFX and PDS.

## Ethics Statement

The study was approved by the local ethics review board of the Medical Faculty of the Ruhr‐University‐Bochum (#4749‐13).

## Conflicts of Interest

The authors declare no conflicts of interest.

## Data Availability

The data that support the findings of this study are available from the corresponding author upon reasonable request.
